# Ecology and phenology of the bat tick *Argas* (*Carios*) *dewae* (Acari: Argasidae)

**DOI:** 10.1017/S0031182024000817

**Published:** 2024-08

**Authors:** Lisa Godinho, Emile van Lieshout, Stephen Griffiths, Mackenzie L. Kwak

**Affiliations:** 1 School of Biosciences, Faculty of Science, The University of Melbourne, Melbourne, Victoria, Australia; 2 School of Agriculture, Biomedicine and Environment, La Trobe University, Melbourne, Victoria, Australia; 3 Department of Disease Control, Faculty of Veterinary Medicine, Hokkaido University, Sapporo, Hokkaido, Japan

**Keywords:** *Argas*, Australia, chiroptera, microbat, parasite, tick, urban

## Abstract

Although 12 soft tick species (Argasidae) are native to Australia, the ecology of most is poorly known. *Argas dewae* parasitizes several insectivorous bat species and has been recorded on humans. Therefore, understanding its ecology is crucial for wildlife health management and public health preparedness. To address this knowledge gap, *A. dewae* populations were monitored from 2 bat hosts (*Chalinolobus gouldii* and *Austronomus australis*) using bat boxes at 3 sites in Victoria, Australia, for 28 months (July 2005–December 2007). A phenological profile undertaken for *A. dewae* revealed that tick load on bat hosts increased throughout winter and peaked in the first month of spring, before collapsing and remaining low throughout the drier late spring and summer periods. There was also further investigation of the relationship between 2 response variables (tick infestation risk and tick load) and a range of explanatory variables (body condition index, sex, age class, bioseason, site, bat density per nest box). In *C. gouldii*, site was the only significant predictor of *A. dewae* infestation risk, while load was correlated with several variables including age class, sex, bioseason, roost density and body condition index. This paper also reports the first records of *A. dewae* from 6 bat species in 3 bat families (Miniopteridae: *Miniopterus australis*; Molossidae: *A. australis*; Vespertilionidae: *Chalinolobus morio*, *Myotis Macropus*, *Vespadelus darlingtonia*, *Vespadelus regulus*) and a second record of *A. dewae* from a human. The first distribution records are presented for *A. dewae* in South Australia, the Australian Capital Territory and Queensland.

## Graphical abstract

**Figure:**
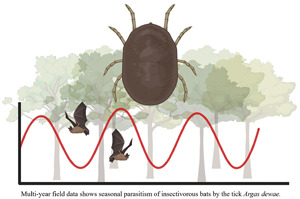

## Introduction

Through their intimate association with hosts, parasites are not only important mediators of natural and sexual selection (Clayton *et al*., 1999; Dougherty *et al*., 2023), but also play important roles in shaping community composition and ecosystem ecology (Hudson *et al*., 2006). Despite arguably representing the most common metazoan lifestyle (Poulin and Morand, 2000), parasites remain one of the most neglected objects of study (Carlson *et al*., 2020), likely due to their size and cryptic habits. As a consequence, relatively little remains known about their distribution and ecology. Yet, such insights may be crucial to a full understanding of the impacts of current anthropogenic environmental change, both on vulnerable host species and ecosystems.

Ticks are specialized ectoparasites that can impose a range of direct costs to reproductive success and survival of their hosts. Tick infestation is often closely associated with reduced host body condition index (BCI) in wild populations (Kwak *et al*., 2023). This relationship may be causal: experimental manipulation under field conditions showed that infestation with tick *Argas cooleyi* could result in up to 65% nestling mortality through anaemia, reductions in body mass and early fledging (Chapman and George, 1991). Indeed, meta-analysis of manipulative studies has shown clear fitness impacts at the population level (Watson, 2013). Other direct fitness costs include paralysis due to toxins (Pienaar *et al*., 2018), damage to the skin, feathers or fur (McLaughlin and Addison, 1986; Buczek *et al*., 2020) and expenditure on host defence such as grooming (Lehmann, 1993). The prevalence and intensity of infestation is often not homogenous: ticks' host preferences may be influenced by factors such as host sex (Kwak *et al*., 2023), age (Jones *et al*., 2015) and reproductive state (Pollock *et al*., 2012; Sándor *et al*., 2019). The optimal level of virulence – the extent to which host fitness is reduced by a parasite – may be relatively high for nest- and roost-inhabiting ticks given their reduced dependence on individual hosts (Lehmann, 1993; but see Watson, 2013).

Ticks can also impose indirect costs as vectors of tick-borne viruses, bacteria and protozoa affecting both humans and wildlife, some of which can be deadly to their hosts (Evans *et al*., 2009; Baneth, 2014; Brackney and Armstrong, 2016; Sarwar, 2017). Numerous pathogenic microbial genera have also been identified in recent years in Australian ticks, including the globally important genus *Borrelia* (i.e. ‘*Candidatus* Borrelia tachyglossi’) (Egan *et al*., 2020). New Australian tick species continue to be discovered too (e.g. *Ixodes woyliei*, *I. healthi* and *I. laridis*) (Ash *et al*., 2017; Heath and Palma, 2017; Kwak *et al*., 2018*a*, 2018*b*). The One Health risks posed by ticks to wildlife and humans are also increasing as novel tick-borne pathogens emerge and geographic range shifts occur in some tick species (Paules *et al*., 2018). Such range shifts can be driven by a variety of ecological factors including habitat changes, host availability and climate (Alkishe *et al*., 2017; Borşan *et al*., 2020). Pathogen spillover between host species is also largely driven by ecological factors (Ostfeld *et al*., 2018). Therefore, studying the relationship between ticks and ecological factors is key to understanding spillover risk between bat species and from ticks to humans.

Two Australian argasids, *Ornithodoros capensis* and *O. gurneyi*, have been recorded parasitizing a wide range of wildlife species and have been reported to cause disease in humans (Kwak, 2018). *Ornithodoros capensis* bites have been associated with blistering and lesions (Humphery-Smith *et al*., 1991). Though little is known about the ecology of the bat tick *Argas dewae*, it has previously been collected from a human (Kaiser and Hoogstraal, 1974). Recently, *Rickettsia japonica* [which is known to cause disease in humans (Mahara, 1997)] was also identified within *A. dewae* (Izzard *et al*., 2018). This suggests that *R. japonica* may act as a pathogen within bat communities, particularly if it moves into naïve populations *via* ticks. It also suggests that *R. japonica* could impact public health in the future if spillover from bats to humans occurred *via A. dewae* as many Australian insectivorous bats roost within man-made structures such as houses (Sanderson *et al*., 2006). However, foundational information about *A. dewae*, such as phenological profiles and host specificity, is needed to construct ecological baselines for bat ticks. Without such data, it is impossible to detect changes in the distribution and phenology of bat ticks, or to understand the ecological drivers which underpin disease risk in bats and humans. Hence, this paper presents key ecological data for *A. dewae* which includes one of the locations [Organ Pipes National Park (OPNP)] at which Izzard *et al*. (2018) identified this species as carrying *R. japonica*.

To elucidate the ecology of *A. dewae*, populations from 2 bat hosts (Gould's wattled bat *Chalinolobus gouldii* and white-striped free-tailed bat *Austronomus australis*) were surveyed. Data were collected from bats roosting in bat boxes at 3 sites across Melbourne, south-eastern Australia, for 28 months (July 2005–December 2007). Both hosts are tree-roosting insectivorous bat species that are common across south-eastern Australia (Churchill, 2008), including in urban areas (Griffiths *et al*., 2020). This study investigated phenological trends to assess the impact of traits of the 2 bat hosts on tick infestation rate and load. In addition, new host records for *A. dewae*, along with new distribution records, are reported.

## Materials and methods

### Study sites

Ticks found on *C. gouldii* and *A. australis* were studied at 3 sites across Greater Melbourne, Victoria, Australia. Gresswell Nature Conservation Reserve (Gresswell NCR), located ~13 km north east of the Melbourne CBD (−37.711461, 145.071491), is a 52 ha river red gum (*Eucalyptus camaldulensis*) grassy woodland. The site is degraded as a result of the surrounding urbanization and has a limited number of large, hollow-bearing trees (Godinho *et al*., 2020). To compensate for the limited number of tree hollows, nest boxes were installed in 1998 by members of the La Trobe University Wildlife Sanctuary (Griffiths *et al*., 2017); 26 of these boxes are of a bat-specific design (Evans and Lumsden, 2011; Godinho *et al*., 2015), and since 2005 have been monitored intermittently as part of larger, ongoing study (Griffiths *et al*., 2020). OPNP, located ~20 km north-west of the centre of Melbourne (−37.669666, 144.770127), is an 85 ha site that was predominately cleared farmland until preserved as a park in 1972, and then revegetated (Edwards, 1974). The majority of trees are <45 years old and lack tree hollows (Bender, 2011). The vegetation is mainly river red gum, with scattered manna gum (*Eucalyptus viminalis*) and an understory of *Acacia* spp. and grasses (Irvine and Bender, 1997). Thirty-seven bat boxes were installed by The Friends of OPNP community group within the riparian zone of Jacksons Creek at OPNP, commencing in 1992, and have since been monitored regularly as part of a larger study (Griffiths *et al*., 2019). Wilson Reserve, located ~9 km east of the centre of Melbourne (−37.779129, 145.046579), is a 35 ha reserve bordered by a golf course, sporting fields and residential housing. Like the other sites, it is dominated by river red gum, with a shrubby understory including *Acacia* spp. Revegetation of this site began in the 1970s, and the *Eucalyptus* trees that were planted have not yet formed hollows. There are, however, several remnant hollow-bearing trees present in the riparian zone (Griffiths *et al*., 2017). Twenty bat boxes were installed by The Friends of Wilson Reserve community group, commencing in 2000, and since 2005 have been monitored regularly as part of a larger study (Godinho *et al*., 2020).

At all 3 sites, any bats occupying the bat boxes were examined on a monthly basis for the presence and abundance of ectoparasites. Monitoring began in winter and spring 2005 at OPNP and Gresswell NCR, respectively, and in spring 2006 at Wilson Reserve. Monitoring ceased at all sites by December 2007. A total of 2148 Gould's wattled bats and 226 white-striped free-tailed bats were sampled for ectoparasites during this period, which included recaptures of marked individuals [Gould's wattled bats – bat bands or Passive Implantable Transponder (PIT) tags (microchips), white-striped free-tailed bats – PIT tags]. Two other bat species were recorded occupying bat boxes, chocolate wattled bat (*Chalinolobus morio*) and large forest bat (*Vespadelus darlingtoni*), however they were not included in this study.

### Identification

During each bat box survey, the number of parasites on each bat host (i.e. the parasite load) captured in a bat box was recorded. Tick prevalence (the proportion of the bat host population carrying the parasite; Bush *et al*., 1997) was calculated. A sample of ticks were removed from bat hosts using tweezers and fixed in 70% ethanol. Species identification was done using a Leica M205C stereomicroscope and morphological characters based on descriptions by Kaiser and Hoogstraal (1974) and Kohls and Hoogstraal (1962). For all bats captured, the individual's sex, age, forearm length and weight was recorded.

### Data analysis

Data were analysed using R version 4.2.1 (R Core Team, 2022). Distributions of macroparasites across hosts follow a count distribution but often are overdispersed due to zero inflation: the majority of parasites are shared amongst a small number of hosts (e.g. Luguterah and Lawer, 2015). Consequently, the data were examined using zero-inflated negative binomial mixture models (ZINB) using the package ‘pscl’ (Zeileis *et al*., 2008). The advantage of these models is that they separately model the processes that lead to excess zeroes, resulting improved inference about the ecological factors that drive individual parasite burdens (i.e. abundance; Rhodes, 2015). The numbers of *A. dewae* ticks found on Gould's wattled bats across the 3 sites were examined. At 2 of the 3 sites (Gresswell National Conservation Reserves and OPNP), bats sampled were individually tagged (with bat bands or microchips) to determine the extent to which individual identity affected parasite burden (see Results section). At Wilson Reserve, individuals were not tagged, so each capture had to be treated as independent.

To assess the influence of repeated measures on re-captured individuals, the correlation of parasite load within individuals across time was measured using an intraclass correlation coefficient (icc) using the package ‘irr’. This value varies from 0 to 1, with 0 indicating larger variation within individuals and lower variation between individuals. A jackknifing approach was used to repeatedly (*n* = 1000) select several observations at random from the individuals with at least that number of captures, which allowed us to examine the mean correlation coefficient with more confidence. This indicated that individual identity was a poor predictor of tick load (see Results section). Consequently, repeated capture data were treated as independent observations in subsequent modelling, obviating the need for mixed-effects models using bat identity as a random factor.

The variables included in our ZINB model were site (Gresswell NCR, OPNP and Wilson Reserve), bioseason [the ‘warm season foraging period’ (WSFP, October–April), during which *C. gouldii* spend most of their time foraging and are rarely in torpor; the ‘torpor period’ (TP, May–August), during which most bats go into torpor and are rarely active; and the ‘post-torpor emergence period’ (PTEP, September), during which bats emerge from their winter torpor and disperse to a range of other roost sites], age (adult or juvenile) and sex of the bats (male or female). Adults were distinguished from juveniles (young of the year) by fusion of the metacarpal–phalangeal joints (Racey, 1974). An interaction between sex and bioseason was also included as significant behavioural differences are likely during different phases of the year, although the interaction was dropped for the logistic component of the model due to lack of significance. Additionally, BCI (using residuals of a least-squares regression of body mass and forearm length based on Schulte-Hostedde *et al*., 2005), and mean monthly maximum temperature were included as covariates. Temperature data represent mean monthly maximum temperatures from the nearest weather station to each site [OPNP: 086282 Melbourne Airport; Gresswell: 086351 Bundoora (Latrobe University); Wilson: 086068 Viewbank]. Post-hoc testing was conducted using the package ‘emmeans’. Male reproductive states were determined by examining the scrotal region for the presence of visible testes (Churchill, 2008). Female reproductive states were determined by examining the nipple for evidence of current or past lactation and gently palpating the abdomen for unborn young (Churchill, 2008). Eight categories, 4 in each sex, were recognized [females LA – lactating, PL – post-lactating, PP – pre-parous or pre-breeding, PX – pregnant; males TA – testes abdominal, TP – testes partially descended, TD – testes fully descended, TR – testes regressed (Churchill, 2008)], but this variable could not be included in the overall model given skew in numbers and confounding over seasons.

### Museum specimens

To assess the host range and distribution of *A. dewae*, one of the authors (M. L. K.) visited the Australian National Insect Collection (ANIC) and examined *A. dewae* specimens held there. New host and distribution records were recorded and are reported in the Results section of this paper.

### Nomenclature

Within this study, we elect to use the classification system and nomenclature for the Argasidae referred to as ‘The American school’ (*senu* Mans *et al*., 2021). Although alternative classification systems have been proposed, we avoid the use of these, as the overall correct classification of the Argasidae remains very unclear and many of these newer systems only serve to complicate and confuse, given that few are based on robust datasets. The most recent of these is by Mans *et al*. (2021) which is based on an extremely small number of loci (mitochondrial genomes and 2 nuclear markers), which do not always provide reliable estimates in deeply diverged ancient clades, like the Argasidae. In comparison, robust phylogenies such as that by Misof *et al*. (2014) for the insects, which utilized 1478 protein-coding genes, provide far more reliable estimates. The study by Mans *et al*. (2021) also included <25% of species in the family Argasidae, and did not adequately correct for the problem of long-branch attraction which is sometimes responsible for erroneous interpretations in phylogenomic trees. Undoubtedly, the nomenclature and classification of the Argasidae will change in coming decades as multiple instances of paraphyly are detected within ‘The American school’ system. However, when the Argasidae are correctly reclassified based on robust phylogenomics utilizing hundreds of genes with good levels of sampling (e.g. 75% of species), we may then adopt such a system. At the present time, studies like that of Mans *et al*. (2021) provide very weak support for major changes to the classification of the Argasidae and as such we choose not to accept this until stronger evidence is provided in support of this system. In this study, we elect to refer to the taxon under examination as *A. dewae* rather than *Carios dewae*.

## Results

### Phenology and ecology

**Figure 1.**
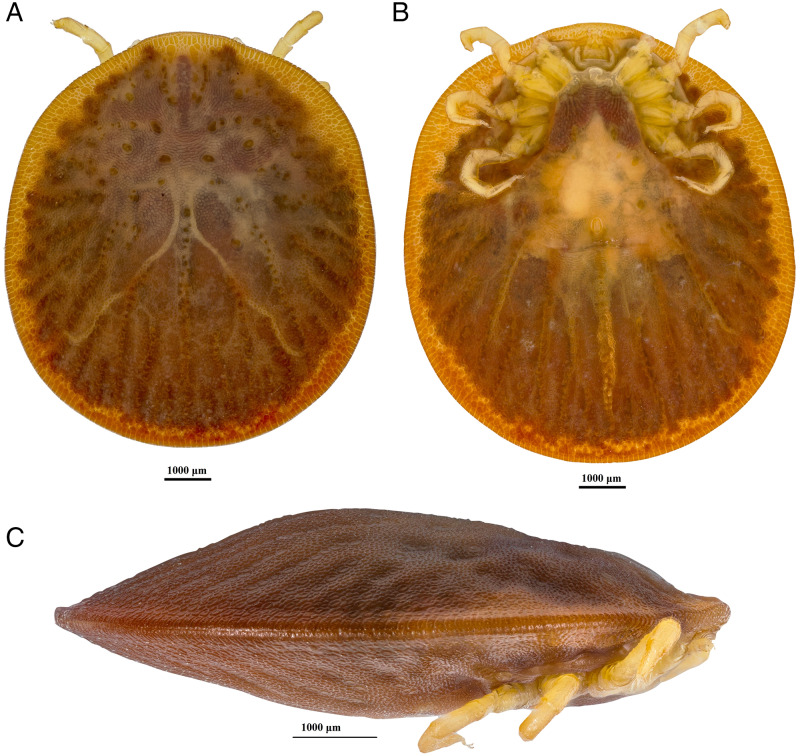
Dew's Australian bat argasid (*Argas dewae*).

*Argas dewae* (Fig. 1) load varied markedly throughout the year, with an increase through autumn and winter and a peak in early spring (September), followed by a rapid decline through late spring and into summer. Although adult *A. dewae* were observed in the bat boxes, it was only larvae and/or nymph stage ticks that were observed attached to hosts and are thereby represented in the data presented. Overall infestation rate was 10.7% in *C. gouldii* and 7.9% in *A. australis* (Table 1).

**Table 1.** Tick prevalence and load on sampled *Chalinolobus gouldii* and *Austronomus australis*

	Individuals sampled	Tick prevalence %	Tick load ± 95% CI
*Chalinolobus gouldii*			
All *C. gouldii*	2417	10.7	4.2 ± 0.80
Adult *C. gouldii*	2149	11.9	4.2 ± 0.81
Juvenile *C. gouldii*	268	1.1	1.3 ± 0.65
Female *C. gouldii*	1642	9.4	4.2 ± 1.11
Male *C. gouldii*	775	13.3	4.1 ± 1.11
*Austronomus australis*			
All *A. australis*	238	7.9	2.1 ± 0.81
Adult *A. australis*	226	8.4	2.1 ± 0.81
Juvenile *A. australis*	12	0.0	0.0 ± 0.00
Female *A. australis*	185	8.1	2.3 ± 0.99
Male *A. australis*	53	7.5	1.0 ± 0.00

**Figure 2.**
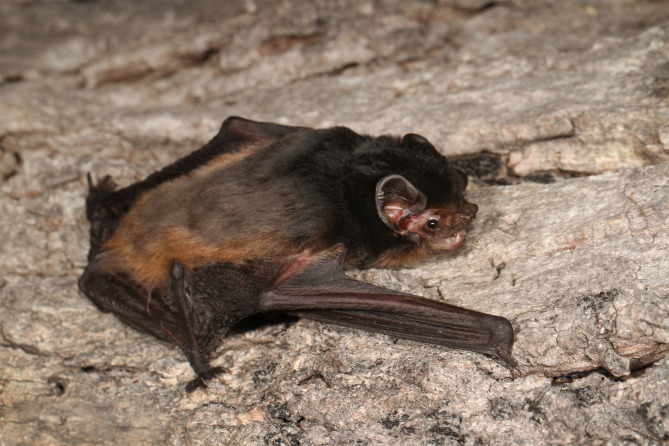
Gould's wattled bat (*Chalinolobus gouldii*).

In total, 2417 observations of the parasite load of *C. gouldii* (Fig. 2) were made; some 11% were juvenile (Table 2). Male and female reproductive states varied strongly across the bioseasons. Bat recapture rates were affected by site: at Gresswell, 146/352 (41.5%) were recaptured, whereas at OPNP only 109/645 (16.9%) were recaptured (*χ*^2^_1_ = 70.744, *P* < 0.001). The mean intraclass correlation coefficient tick load within individuals that had been captured between 2 and 10 times was 3.63%, justifying the use of tick load data from recaptured bats as independent data points.

**Table 2.** Sample sizes of adult and juvenile *C. gouldii* bats across the 3 bioseasons for the 3 sampled sites

Age	Torpor period (May–Aug)			Post-torpor emergence period (Sep)			Warm season foraging period (Oct–Apr)		
	Gr	OP	WR	Gr	OP	WR	Gr	OP	WR
Adult	104	253	65	129	138	51	482	672	255
Juvenile	0	0	0	0	0	0	40	173	55

Gr, Gresswell National Conservation Reserves; OP, Organ Pipes National Park; WR, Wilson Reserve.

**Figure 3.**
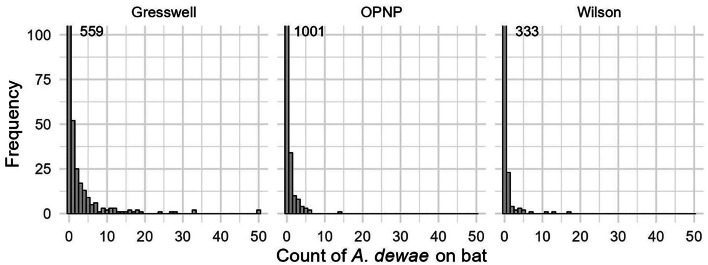
Histograms for counts of *A. dewae* ticks on *C. gouldii* bat across the 3 sites sampled. Note that captures with zero tick counts exceed the *y*-axis range and exact numbers are indicated in the graph.

**Figure 4.**
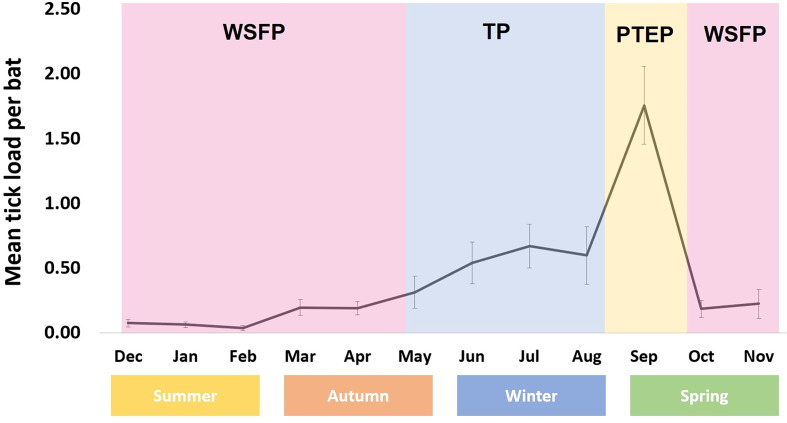
Phenological map of *Argas dewae* annual abundance on its bat hosts (errors bars denote standard error).

**Figure 5.**
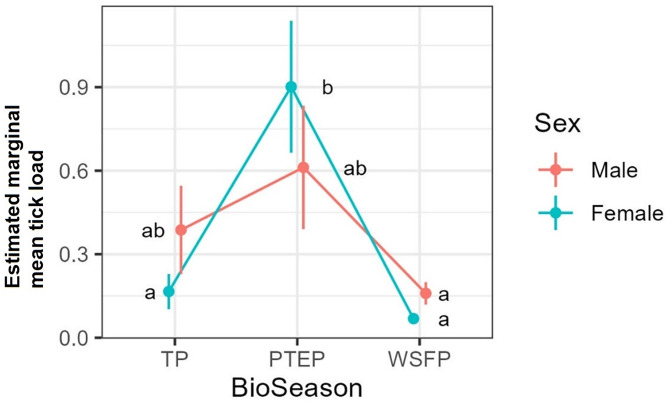
Estimated marginal mean tick loads on Gould's wattled bats by sex over the 3 bioseasons (TP, torpor period; PTEP, post-torpor emergence period; WSFP, warm season foraging period). Values are averaged across sampled sites and ages. Different letters indicate significant differences following Sidak post-hoc pairwise comparisons at *α* = 0.05.

**Figure 6.**
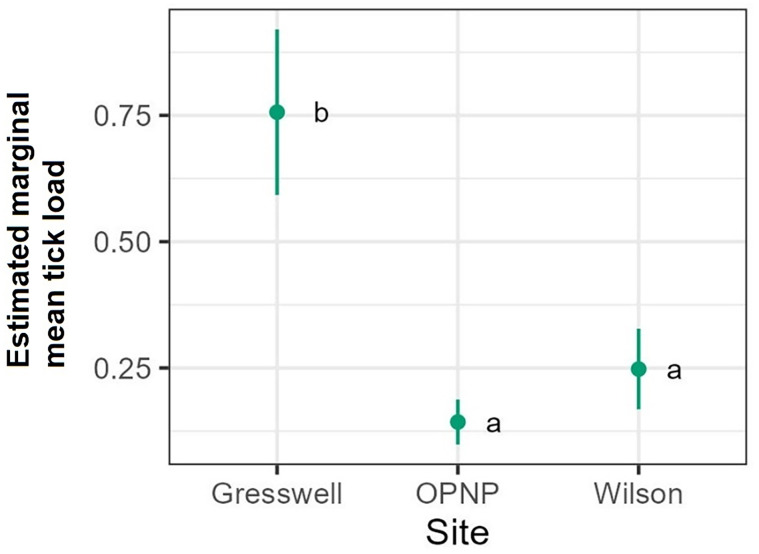
Estimated marginal mean tick loads on Gould's wattled bats across the 3 sampled sites Values are averaged across sexes, ages, and bioseasons. Different letters indicate significant differences following Sidak post-hoc pairwise comparisons at *α* = 0.05.

**Figure 7.**
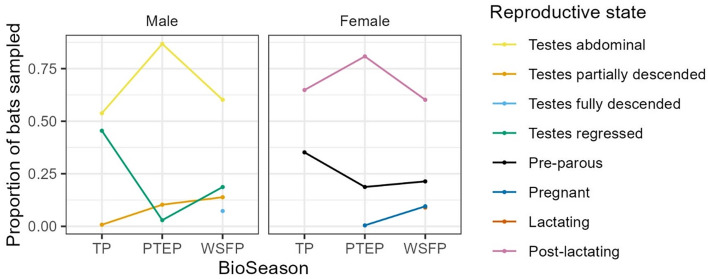
Changes in male and female reproductive states over the bioseasons (TP, torpor period; PTEP, post-torpor emergence period; WSFP, warm season foraging period). Note that the lactating and pregnant female data points largely overlap in the WSFP.

The tick count data were highly over-dispersed relative to a Poisson distribution (variance exceeded the mean, see Table 3). Indeed, the distribution of numbers of *A. dewae* counted was highly biased towards zero across all sites (Fig. 3). Our ZINB model explained a moderate amount of the total variation in tick load (Nagelkerke pseudo *R*^2^: 0.31). The count component of the model indicated that tick abundance was influenced by an interaction of sex and bioseason (Table 4). This interaction was evident in the estimated marginal mean tick loads of the overall model: males and females during the warm season form 1 group with females in the torpor season, with distinctly lower tick loads than females in the post-torpor emergence season, while the remaining groups (males in the torpor and post-torpor emergence season) having tick loads similar to both aforementioned groups (Figs 4 and 5). Additionally, tick load differed between sites: bats from Gresswell had higher tick loads than those from OPNP and Wilson reserve (see Fig. 6). Tick abundances were lower in juveniles and in boxes with greater numbers of bats (see Table 4). In contrast, the sole factor that influenced the probability of zero tick loads was site, with the lowest probability found in Gresswell (see Table 4, Fig. 3). Additionally, greater body condition indices were associated with greater probabilities of zero tick loads (Table 4). Male and female reproductive states varied strongly across the year and were confounded by bioseason, thus making it infeasible to analyse the impact of reproductive variables on tick infestation (Fig. 7).

**Table 3.** Descriptive statistics for counts of *A. dewae* ticks on adult Gould's wattled bat hosts at the 3 sites in Melbourne, Australia

Site	*N*	Minimum	Maximum	Mean	Variance
Gresswell NRC	715	0	50	1.17	18.5
Organ Pipes NP	1063	0	14	0.127	0.505
Wilson Reserve	371	0	17	0.288	2.03

**Table 4.** Zero-inflated negative binomial (ZINB) mixture modelling results for *A. dewae* tick load on *C. gouldii*. Bold values indicate statistically significant *p* values.

	Log odds ± s.e.m.	*χ*^2^	d.f.	*P*
Count (abundance)				
Site (OPNP, Wilson)	−0.80 ± 0.30, −0.62 ± 0.31	8.70	2	**0**.**013**
Bioseason (PTEP, WSFP)	0.46 ± 0.52, −0.65 ± 0.69	28.67	2	**5.95 × 10^−7^**
Sex (female)	−0.80 ± 0.32	4.76	1	**0.029**
Bioseason × sex		8.71	2	**0**.**013**
Age (juvenile)	−3.14 ± 0.88	12.87	1	**0**.**0003**
BCI	0.19 ± 0.33	0.34	1	0.560
Temperature	0.03 ± 0.07	0.20	1	0.657
Bats in box	−0.04 ± 0.01	8.71	1	**0**.**003**
Zero inflation				
Site (OPNP, Wilson)	1.50 ± 0.33, 0.92 ± 0.36	13.11	2	**0**.**001**
Bioseason (PTEP, WSFP)	0.01 ± 0.49, 0.45 ± 0.70	0.82	2	0.665
Sex (female)	0.09 ± 0.29	0.09	1	0.764
Age (juvenile)	−13.83 ± 835.77	1.49	1	0.222
BCI	1.14 ± 0.39	7.55	1	**0**.**006**
Temperature	0.12 ± 0.06	3.014	1	0.083
Bats in box	−0.02 ± 0.02	1.46	1	0.227

Model output is split into abundance and binomial components, which separately describe the influence of predictors on non-zero tick loads and the likelihood of zero tick loads, respectively. Estimates for site and bioseason are relative to reference levels (Gresswell and TP, respectively).

### Host interactions and distribution

**Figure 8.**
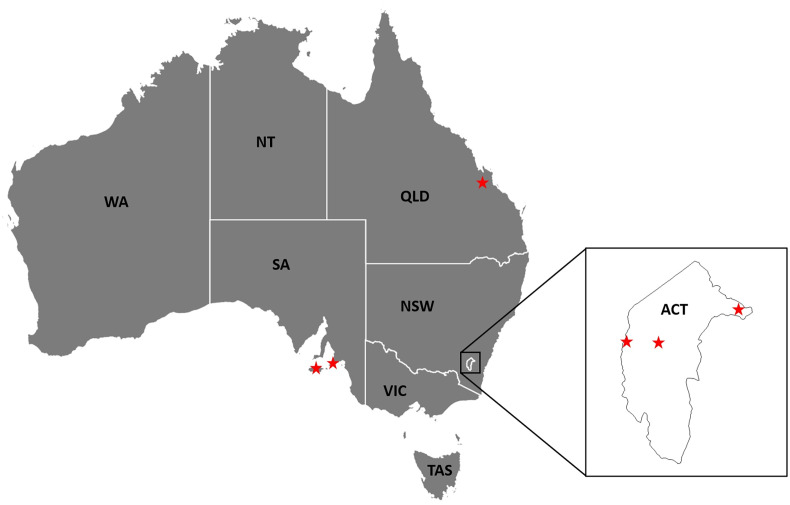
New state and territory records for *Argas dewae* in Australia (QLD, Queensland; NSW, New South Wale; VIC, Victoria, ACT, Australian Capital Territory; TAS, Tasmania; SA, South Australia; WA, Western Australia; NT, Northern Territory).

*Argas dewae* has been recorded from 7 different host species in past publications (Table 5). Based on specimens at the ANIC, records are presented from 6 new hosts of *A. dewae*, namely *A. australis*, *C. morio*, large-footed Myotis (*Myotis macropus*), large forest bat (*V. darlingtoni*), southern forest bat (*Vespadelus regulus*) and little bent-wing bat (*Miniopterus australis*) (Table 6). These new host records also represent the first records of *A. dewae* from the 2 bat families Molossidae (*A. australis*) and Miniopteridae (*M. australis*). Another notable interaction record based on museum specimens was that of *A. dewae* crawling on a human at Batemans Bay (NSW) on 24 December 1975 (Table 6). In addition, this study includes the first records of *A. dewae* from the 3 new states/territories: Queensland, Australian Capital Territory, South Australia (Table 7, Fig. 8).

**Table 5.** Hosts of Dew's bat tick (*Argas dewae*) reported in the literature

Host family	Host species	Common name	Reference
Vespertilionidae	*Chalinolobus dwyeri*	Large-eared pied bat	Kaiser and Hoogstraal (1974)
Vespertilionidae	*Chalinolobus gouldii*	Gould's wattled bat	Kaiser and Hoogstraal (1974)
Vespertilionidae	*Falsistrellus tasmaniensis*	Eastern false pipistrelle	Kaiser and Hoogstraal (1974)
Vespertilionidae	*Nyctophilus geoffroyi*	Lesser long-eared bat	Kaiser and Hoogstraal (1974)
Vespertilionidae	*Vespadelus vulturnus*	Little forest bat	Kaiser and Hoogstraal (1974)
Rhinolophidae	*Rhinolophus megaphyllus*	Smaller horseshoe bat	Kaiser and Hoogstraal (1974)
Hominidae	*Homo sapiens*	Human (child in pram)	Kaiser and Hoogstraal (1974)

**Table 6.** New host records for Dew's bat tick (*Argas dewae*)

Host family	Host species	Common name	ANIC reference code
Molossidae	*Austronomus australis*	White-striped free-tailed bat	48 005 284
Vespertilionidae	*Chalinolobus morio*	Chocolate wattled bat	48 005 281
Vespertilionidae	*Myotis macropus*	Large-footed myotis	48 005 286
Vespertilionidae	*Vespadelus darlingtoni*	Large forest bat	48 005 268
Vespertilionidae	*Vespadelus regulus*	Southern forest bat	48 005 266
Miniopteridae	*Miniopterus australis*	Little bent-wing bat	48 000 136
Hominidae	*Homo sapiens*	Human (crawling on clothes)	48 000 139

ANIC, Australian National Insect Collection.

**Table 7.** New state/territory records within Australia for *Argas* (*Carios*) *dewae*

State/territory	Location	Host species	Date	ANIC reference code
ACT	Kowen Forest	*Chalinolobus gouldii*	N/A	48 005 265
ACT	Gibraltar Falls	*Vespadelus darlingtoni*	23 Mar 1987	48 005 269
ACT	Mount Franklin	*Nyctophilus geoffroyi*	N/A	48 005 272
QLD	The Caves	*Miniopterus australis*	26 Jul 1974	48 000 136
SA	Kangaroo Island (Karatta)	*Vespadelus regulus*	14 Apr 1987	48 005 266
SA	Deep Creek Conservation Park	*Nyctophilus geoffroyi*	16 Apr 1987	48 005 280

ACT, Australian Capital Territory; SA, South Australia; QLD, Queensland; N/A, not available.

## Discussion

### Phenology of *A. dewae*

In our study, the abundance of *A. dewae* was correlated with seasonal activity patterns of its host *C. gouldii*. As bats in this species enter torpor during winter in southern Australia, they become largely inactive and only rewarm during ephemeral mild weather events (Dixon and Huxley, 1989; Churchill, 2008), probably in relation to temporary increases in insect abundance (Turbill, 2006, 2008). Since grooming requires arousal from torpor, the progressive increase in abundance of ticks throughout the cooler autumn and winter months suggests grooming is markedly reducing during this period. As the main torpor period ends for *C. gouldii* in early spring, the bats tend to form large groups in bat boxes (e.g. up to 58 bats; Godinho *et al*., 2020). This type of clustering behaviour likely elevates temperatures inside bat boxes containing many bats (Willis and Brigham, 2007). The combination of high host density and higher roost temperatures may facilitate higher tick numbers due to the abundance of food, which facilitates higher metabolic rates (and likely faster growth rates) in the poikilothermic ticks. Following the PTEP, ambient temperatures continued to rise at the onset of the WSFP, and the abundance of ticks markedly declined. This decline may be due to increased grooming activity by the bat hosts, as the abundance of their insect prey during the warmer spring and summer months allows them to meet the energetic demands needed to fuel frequent grooming (Godinho *et al*., 2013). Although *C. gouldii* use winter torpor in the southern part of their range (Stawski and Currie, 2016), in the northern reaches of their distribution, they are generally active all year and are not torpid during the winter (Chruszcz and Barclay, 2002). It is unclear whether the phenological patterns observed for *A. dewae* in the south mirror those in the northern populations. In other tick species, climate has been shown to have a marked effect on tick activity (Qviller *et al*., 2014). Therefore, future studies should seek to address this, as the phenology of *A. dewae* may be more flexible than this study suggests.

Although there was no direct effect of host sex on tick load and prevalence, the phenological patterns of *A. dewae* load did depend on sex: with a significant spike in tick load detected during PTEP only in female bats. The effect of sex on tick load and prevalence in bats is variable but tends to be male biased (Piksa *et al*., 2014; Kwak *et al*., 2023; but see: Sándor *et al*., 2019). This general pattern is consistent with theory that predicts that the expression of male secondary sexual characteristics signals the ability to resist parasites (Balenger and Zuk, 2014) and that the magnitude of this difference should depend on the degree of sexual selection in a species (Moore and Wilson, 2002). Gould's wattled bats show little sexual dimorphism beyond that in overall size in favour of females (Tidemann, 1986) as is common across bats (Muñoz-Romo *et al*., 2021), although little research effort has been dedicated to this species in this regard. The exact reason for the discrepant phenology of tick load between the sexes remains unclear but could be related to differences in immune function and condition during this period.

### Infestation ecology of bat ticks

Although higher parasite loads are often associated with high host densities in roosts (e.g. Christe *et al*., 2007; Sharifi *et al*., 2008), in this study they were not. Individual *C. gouldii* often utilize large numbers of different roosting sites across their range (Godinho *et al*., 2015, 2020). This tendency allows ticks to disperse across the range of the individual, preventing tick numbers from building up within any single roost. While solitary bats are vulnerable to accumulating large numbers of nidicolous ectoparasites such as *A. dewae*, particularly when the roost has not been visited frequently, gregarious species may dilute individual parasite loads when parasites can switch hosts. This may explain why lower tick loads were correlated with nest box which had higher numbers of bats. The lower tick prevalence and loads in juvenile bats may be explained by the fact that they are much more abundant during the WSFP, during which tick abundance is far lower than in the PTEP or WHP. Juvenile bats are also commonly encountered with their mothers, which may also act to dilute the ticks present in the roost.

In correlational studies of parasite load, low body condition is often assumed to increase susceptibility to parasites or, inversely, to result from infestation. However, causality in the relationship between parasite load and condition is difficult to ascertain (Coulson *et al*., 2018). In fact, the relationship between these variables can range from positive to negative and may be due to the indirect effects of other environmental factors (Amo *et al*., 2007; Sanchez *et al*., 2018). In this study, higher BCI values were associated with greater probabilities of zero tick load, although it should be noted that other variables such as bioseason and site were much more strongly associated with risk of infestation. This suggests that, while BCI may have some bearing on tick–host ecology, other factors may act as more important determinants of tick load in *C. gouldii*. It is therefore suggested that further studies which manipulate tick load are necessary in this species to reveal the underlying relationships that parasite load and presence have with factors such as host condition, behaviour, habitat and season.

### Distribution and hosts

*Argas dewae* is primarily a parasite of insectivorous bats and appears to tolerate both cave and tree hollow roosting habits in its hosts. Based on previously published records (Kohls and Hoogstraal, 1962; Kaiser and Hoogstraal, 1974), as well as those newly reported here, *A. dewae* has been collected from 12 bat species from 4 bat families. It has also been found on humans on 2 occasions, though the extent of feeding is unclear, and humans clearly represent atypical interactions.

In this study, the results indicate that the probability of bats carrying any *A. dewae* ticks can vary significantly between relatively proximate populations. The exact reasons for these between-population differences are unclear but can be associated with both host and environmental factors (Ruiz-Fons *et al*., 2012). *Argas dewae* is distributed across most of eastern Australia from as far west as South Australia (Kangaroo Is.) and south into Tasmania, then extends through Victoria, the Australian Capital Territory, New South Wales and into Queensland to just north of Rockhampton (Fig. 8). Distribution records for *A. dewae* are sparse owing to relatively limited research on the Australian native Argasidae and the specialized ecology of the tick which makes sampling challenging. It is unclear whether the range of *A. dewae* extends into the Northern Territory or Western Australia, as one of its primary hosts, *C. gouldii*, also occurs in both of these regions (Churchill, 2008). Although a number of the hosts of *A. dewae* have ranges which extend into New Guinea (e.g. *M. australis*) (Kitchener and Suyanto, 2002), it is unknown whether the range of *A. dewae* also extends outside of Australia. The lesser oriental bat argasid (*Argas pusillus*) has been recorded on insectivorous bats north of Australia, in New Guinea, but *A. dewae* has not been reported (Hoogstraal, 1982). This may suggest that *A. pusillus* is occupying the niche that *A. dewae* fills in Australia and that competitive exclusion has prevented *A. dewae* from becoming established in this region.

### One Health risk

Phenological data on vectors are important when formulating public health policy or undertaking surveillance efforts. In southern Australia, September may represent a period of higher risk of zoonotic spillover for *A. dewae*, and potentially also *R. japonica* from this tick. During the PTEP, ticks are significantly more abundant than at any other time of year, corresponding to increased activity of their hosts. The main bat species at the 3 peri-urban habitats sampled in this study, *C. gouldii*, was the dominant host of *A. dewae*. *Chalinolobus gouldii* is well adapted to living in urbanized habitats and is known to roost in ceilings and basements (Dixon and Huxley, 1989; Chruszcz and Barclay, 2002). Therefore, *C. gouldii* may bring *A. dewae* into homes from which they may parasitize humans and transmit *R. japonica*. Notably, interactions between humans and *A. dewae* to date have rarely been recorded (but see Kaiser and Hoogstraal, 1974). However, shifts and changes in tick or host ecology driven by habitat loss or climate change may increase the likelihood of human–bat–tick interactions and result in future spillover events. To better prepare for such possibilities, more extensive bio-surveillance efforts should be directed at elucidating the microbiomes of both *A. dewae* and *C. gouldii*; as well as the extent to which *A. dewae* and *C. gouldii* cohabitate with humans.

## Data Availability

The data that support the findings of this study are openly available in figshare at http://dx.doi.org/10.26188/27321879.
